# An Epidemiologic Analysis of Co-Occurring Alcohol and Drug Use and Disorders: Findings From the National Epidemiologic Survey of Alcohol and Related Conditions (NESARC)

**Published:** 2008

**Authors:** Daniel Falk, Hsiao-ye Yi, Susanne Hiller-Sturmhöfel

**Keywords:** National Epidemiologic Survey on Alcohol and Related Conditions (NESARC), alcohol use, drug use, alcohol use disorders (AUDs), drug use disorders (DUDs), co-morbidity, prevalence, epidemiology, racial/ethnic differences, gender differences

## Abstract

The 2001–2002 National Epidemiologic Survey on Alcohol and Related Conditions (NESARC) sought to determine the prevalence of alcohol use and alcohol use disorders (AUDs), other drug use and drug use disorders (DUDs), and co-use and co-morbidity in the general adult U.S. population. Findings indicate that 5.6 percent of U.S. adults used both alcohol and drugs in the past year and that 1.1 percent had a co-morbid AUD and DUD. Alcohol use prevalence peaked between the ages of 25 and 44 and declined thereafter. The prevalence of other drug use, co-use, AUDs, DUDs, and co-morbid disorders was highest between the ages of 18 and 24 and declined steadily thereafter. Women and men showed similar trends for alcohol use, drug use, and co-use. Among ethnic/racial groups evaluated, Whites displayed the highest rates of alcohol use and American Indians/Alaskan Natives the highest rates of drug use. For AUDs, DUDs, and co-morbid disorders, rates were highest among American Indians/Alaskan Natives. The prevalence of drug use, weekly drug use, and DUDs increased with increasing levels of alcohol consumption and the presence of AUDs. The proportion of people with AUDs who had a co-morbid DUD varied considerably by drug type. These findings have important implications for the development of prevention and intervention approaches.

Numerous epidemiological studies (Grant et al. 2004) have demonstrated that alcohol use disorders (AUDs) (i.e., alcohol abuse and alcohol dependence) are widespread in the general population of the United States, with approximately 8.5 percent of adults having had an AUD in the past year. Importantly, many people suffering from AUDs also suffer from one or more other psychiatric disorders, including other drug use disorders (DUDs), mood disorders (e.g., major depression), anxiety disorders, or personality disorders (e.g., antisocial personality disorder). This co-morbidity has important implications for diagnosis and, particularly, therapy because people suffering from an AUD and other co-morbid disorders may not respond as well to alcoholism treatment as people with only an AUD. (For more information on treatment of patients with co-morbid AUDs and DUDs, see the article by Arias and Kranzler, pp. 155–167.)

In general, the term co-morbidity refers to the co-occurrence of two or more psychiatric disorders. Recently, however, researchers have begun to distinguish between two types of co-morbidity, based on the disorders involved (Angold et al. 1999):

- Homotypic co-morbidity, which refers to the co-occurrence of disorders from the same diagnostic group, such as the co-occurrence of substance use disorders like AUDs and DUDs.
- Heterotypic co-morbidity, which refers to the co-occurrence of disorders from different diagnostic groupings, such as the co-occurrence of an AUD with a mood or anxiety disorder.

Over the past two to three decades, researchers have intensely studied the relationships between AUDs and heterotypic psychiatric disorders to determine the prevalence and underlying causes of the co-morbidity (for a review, see Petrakis et al. 2002). Much less is known about the homotypic co-morbidity of AUDs and DUDs, the potential causes or mechanisms underlying this co-morbidity, and its impact on treatment seeking and treatment outcome. The existing studies nonetheless have provided valuable information on homotypic co-morbidity.

Some of this research has focused on the possible mechanisms underlying the concurrent use of alcohol and other drugs. For example, researchers have investigated the possibility of genetic and environmental risk factors that may account for part of the observed association. (For more information, see the article by Dick and Agrawal, pp. 111–118.) Other studies have examined the neurobiological aspects of co-morbid alcohol and other drug use, seeking to identify shared mechanisms through which the different drug classes may act on the brain. (For more information, see the article by Cruz et al., pp. 137–147.)

Although these avenues of research are of the utmost importance, it is equally critical to obtain a solid understanding of the scope of the problem. Thus, it is important to know the rates of AUDs, DUDs, and co-morbid disorders in the general population and whether certain demographic subgroups are particularly at risk of having both disorders.

To date, however, only a handful of large epidemiological studies (Burns and Teesson 2002; Grant and Pickering 1996; Helzer and Pryzbeck 1988; Kessler et al. 1997; Regier et al. 1990; Ross 1995; Stinson et al. 2005) have investigated the prevalence of AUD and DUD co-morbidity in the general population. In general, these studies found that between 13 and 17 percent of individuals with a past-year AUD also had a co-morbid past-year DUD. On a lifetime basis, the co-morbidity rates are higher. For instance, among individuals with a lifetime AUD, rates of a lifetime DUD ranged from 20 to 23 percent. Furthermore, lifetime co-morbid DUD rates were relatively higher among respondents with alcohol dependence than among those who abuse alcohol (Kessler et al. 1997; Ross 1995) and among women than men (Kessler et al. 1997).

Thus, these studies have demonstrated that AUDs and DUDs are quite likely to co-occur. However, despite this important insight, these studies suffer from several drawbacks that make it difficult to accurately determine the extent of co-morbidity. First, all but a few of the studies (Burns and Teeson 2002; Stinson et al. 2005) reported prevalence of alcohol and other drug use and/or co-morbidity on a lifetime basis rather than on a current (i.e., 12-month) basis. Data derived from lifetime diagnoses, however, produce larger co-morbidity estimates than data derived from past-year estimates. This is because lifetime co-morbid diagnoses include additional cases in which AUDs and DUDs were not present contemporaneously but rather may have been separated by a considerable time gap, even several years apart.^1^

Second, with a few exceptions (Grant and Pickering 1996; Helzer and Pryzbeck 1988; Stinson et al. 2005), most co-morbidity studies have not systematically compared the prevalence of drug-specific DUDs among people with an AUD. Disaggregation of the DUDs into specific types (e.g., cannabis, cocaine, opioids, heroin, and prescription medications), however, is warranted because the prevalence of specific DUDs has been shown to vary greatly among the population of people with comorbidities as well as in the general population (Stinson et al. 2005).

Third, most of the existing co-morbidity analyses did not present information on broader alcohol and drug co-use, which also includes people who do not meet the diagnostic criteria of an AUD or DUD. A few recent epidemiological studies have examined the extended scope of co-use in the United States. These studies generally have focused on co-use of one class of drugs, such as cannabis use (Degenhardt and Hall 2003), rather than presenting data that include all types of drug use. Despite this restricted focus, data from these studies have suggested an interesting dose-response relationship between increasing levels of alcohol consumption and the increased prevalence of specific types of drug use.

In a recent study, Stinson and colleagues (2005), using data from the 2001–2002 National Epidemiologic Survey on Alcohol and Related Conditions (NESARC), described the rates of drug-specific disorders among individuals with AUDs as well as sociodemographic characteristics associated with co-morbid AUDs and DUDs. The present article expands on this work by Stinson and colleagues (2005) and fills some gaps in previous studies. The main objective is to present detailed epidemiologic information on the prevalence of homotypic alcohol and drug co-use and co-morbidity in the U.S. adult population. The analysis is based on comprehensive definitions of drug use and DUDs and includes separate analyses for 10 classes of drugs, with most prevalence estimates presented by gender, age, and race/ethnicity. In addition, this study explores how the prevalence of drug use and DUDs varies with levels of alcohol consumption and the presence of AUDs. Also, rates of drug-specific drug use and DUDs among adults with AUDs are provided.

## Methods

### Data

The present study is based on data from the first wave of NESARC, conducted by the National Institute on Alcohol Abuse and Alcoholism (NIAAA) in 2001–2002. NESARC is among the largest nationally representative co-morbidity surveys ever conducted and includes extensive questions on alcohol and drug use and related disorders. The NESARC sample includes 43,093 respondents ages 18 and older, representing the civilian, noninstitutionalized adult population of the United States, including residents of all 50 States and the District of Columbia. Military personnel living off base and persons living in noninstitutionalized group-quarter housing, such as boarding houses, shelters, and dormitories, also were included (Grant et al. 2003). The sampling frames for housing units and group-quarter units were derived from the Census 2000/2001 Supplementary Survey and the Census 2000 Group Quarters Inventory, respectively. NESARC oversampled Blacks, Hispanics, and young adults (ages 18–24) to allow for more reliable estimates of these groups. Data were collected via face-to-face, computer-assisted interviews in household settings. From each household, one adult was selected to be interviewed. The overall response rate for NESARC was 81 percent.

### Measures

#### Alcohol Measures

All alcohol measures employed in the present analysis reflect consumption and diagnostic status during the past year (i.e., the 12 months preceding the time of the NESARC interview). Although NESARC collected alcohol data separately for each of four beverage types (i.e., wine, beer, coolers, and distilled spirits), the results presented here are the aggregate across beverage types. The various alcohol measures are defined as follows:

- Lifetime abstainer—Never had one or more drinks of alcohol during the course of a lifetime, excluding small tastes or sips.
- Former drinker—Had at least one drink prior to the past year but no drinks during the past year.
- Current drinker (or alcohol user)— Had at least one drink of alcohol during the past year.

Based on past-year consumption, current drinkers are divided into three drinking levels:^2^

– *Light drinker—*Had three or fewer drinks per week.
– *Moderate drinker—*Had 4–14 drinks per week for men and 4–7 drinks per week for women.
– *Heavy drinker—*Had more than 14 drinks per week for men and more than 7 drinks per week for women.

- AUD—Met the criteria articulated in the Diagnostic and Statistical Manual of Mental Disorders, Fourth Edition (DSM–IV) (American Psychiatric Association 1994) for alcohol abuse and/or alcohol dependence during the past year. AUDs were diagnosed using NIAAA’s *Alcohol Use Disorder and Associated Disabilities Interview Schedule* DSM–IV Version (AUDADIS–IV) (Grant et al. 2001), an interview instrument based on the DSM–IV criteria. Diagnoses of AUDs in NESARC are defined as follows:
  – *Alcohol abuse—*Met at least one of the following four criteria for DSM–IV alcohol abuse (and did not meet criteria for alcohol dependence): continued use despite social or interpersonal consequences, hazardous use, alcohol-related legal consequences, or neglect of role responsibilities in favor of drinking.
  – *Alcohol dependence—*Met at least three of the following seven criteria for DSM–IV alcohol dependence in the same 12-month period: tolerance, withdrawal syndrome or drinking to relieve/ avoid withdrawal, impaired control over drinking, persistent desire or unsuccessful attempts to cut down or stop drinking, much time spent drinking, reducing/giving up important activities in favor of drinking, and continued drinking despite physical or psychological problems caused by drinking.

#### Drug Measures

Like the alcohol measures above, the drug measures employed in the present study also reflect past-year use and diagnostic status. NESARC collected data separately for 10 classes of drugs: cannabis, cocaine (including crack cocaine), opioids^3^ (other than heroin or methadone), hallucinogens, amphetamines, tranquilizers, sedatives, heroin,^4^ inhalants/solvents, and other drugs. Note that nicotine was not included among these drug classes because the discussion in this article focuses on co-occurrence of alcohol use with illicit drug use.^5^

The various drug measures are defined as follows:

- *Drug use—*Had used at least 1 of the 10 classes of drugs during the past year.
- *Weekly drug use—*Had used at least 1 of the 10 classes of drugs during the past year at least once every week.
- *DUD—*Met DSM–IV criteria for drug abuse and/or drug dependence during the past year for at least 1 of the 10 classes of drugs. All drug-specific diagnoses of abuse and dependence were derived using the same algorithm and were aggregated to produce the measure of any DUD. DSM–IV criteria for the abuse and dependence of specific drugs are similar to those associated with alcohol. However, the withdrawal syndrome, as part of the criteria for dependence, is only applicable to certain classes of drugs (i.e., sedatives, tranquilizers, opioids, amphetamines, and cocaine).

#### Co-Occurrence of Alcohol and Drug Measures

Based on past-year alcohol and drug use and disorder status, two measures are defined:

- *Co-use—*Had used both alcohol and drugs within the past year.
- *Co-morbid—*Had both an AUD and a DUD in the past year.

### Data Analysis

Data presented in this article are descriptive in nature. All prevalence estimates of alcohol and drug use and disorders are presented as percentages of the total adult population in the United States or of a subpopulation group. All analyses are carried out for men and women separately, except for rates of disorders by age and race/ethnicity, which are presented for both genders combined because of small sample sizes. Rates of alcohol and drug use, co-use, disorders, and co-morbidity are estimated for four age-groups (ages 18–24, 25–44, 45–64, and 65 and older) and five mutually exclusive racial/ethnic groups (i.e., White, Black, American Indian/Alaskan Native, Asian/Native Hawaiian/Pacific Islander, and Hispanic). To demonstrate the relationship between drinking characteristics and drug use, the prevalence of drug use, weekly drug use, and DUDs are presented by drinking categories (i.e., lifetime abstainer, former drinker, and current drinker). In addition, current drinkers are further divided into five mutually exclusive subgroups (i.e., light drinking, moderate drinking, heavy drinking, alcohol abuse, and alcohol dependence). Drug-specific co-morbidity rates are shown as the prevalence of specific drug use and DUDs among those respondents with any AUD. All estimates were weighted by sampling weights to represent the entire adult population of the United States. Standard errors^6^ for all estimates were generated using the software package SUDAAN (Research Triangle Institute 2004), which takes into account the effect of the NESARC complex sampling design. Based on the standard errors, confidence intervals at the 95 percent level are produced and presented for all estimates to help readers make comparisons between estimates. In addition, estimates with a relative standard error greater than 0.3 are considered unreliable and are flagged by an asterisk. Relative standard error is a measure of the unreliability of the estimate, calculated by dividing the standard error by the value of the estimate (Klein et al. 2002).

## Results

### Prevalence of Alcohol and Drug Use and Co-Use

According to the NESARC data, approximately 65 percent of adults in the United States used alcohol, whereas only about 6 percent used drugs during the past year (see table 1). Furthermore, almost 6 percent of adults used both alcohol and drugs, representing approximately 12.6 million adults in the U.S. population. These data indicate that most drug users (approximately 90 percent) used alcohol, whereas only a small minority of alcohol users (less than 9 percent) used drugs. Although men and women had similar rates of using drugs only (0.7 and 0.6 percent, respectively^7^), men were much more likely than women to use alcohol only (64.7 vs. 55.4 percent, respectively^8^). Moreover, men were more likely to use both alcohol and drugs, whereas women were more likely than men to abstain from both substances.

#### Prevalence by Age-Group

**Figure 1:**
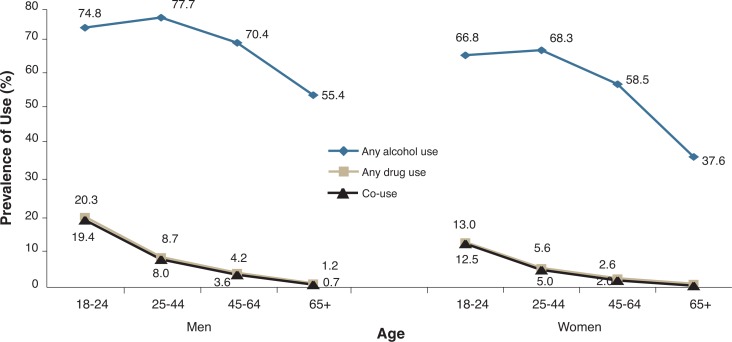
Prevalence (%) of past-year alcohol use, drug use, and co-use by age and gender in the United States, 2001–2002 NESARC. NOTE: Data are drawn from Table 1.

The prevalence of alcohol use by age-group showed a somewhat curvilinear pattern for both men and women (figure 1). Alcohol use already was common among people ages 18–24, with approximately 75 percent of men and approximately 67 percent of women in that age-group using alcohol. Alcohol use peaked at slightly higher levels at ages 25–44, began to decline among people ages 45–64, and was lowest among people ages 65 and older, among whom slightly more than half of men and slightly more than one-third of women used alcohol.

In contrast, drug use declined monotonically with age for both genders, with the highest prevalence among the youngest group and the lowest prevalence among the oldest group. Because most drug users also used alcohol, the trajectory of co-use across age-groups is very similar to that of drug use—the highest rates were found among people ages 18–24 (almost one-fifth for men and one-eighth for women) and the lowest rate among people ages 65 and older (less than 1 percent for both genders).

#### Prevalence by Race/Ethnicity

The prevalence of alcohol use varied among racial/ethnic groups for both men and women. For both genders, Whites displayed the highest rates (about 75 percent for men and about 65 percent for women) and Asians/Native Hawaiians/Pacific Islanders displayed the lowest rates (about 62 percent for men and about 36 percent for women). The prevalence of drug use for both genders was highest among American Indians/Alaskan Natives and lowest among Asian/Native Hawaiian/ Pacific Islander men and Hispanic women. Parallel to this pattern, the prevalence of using both alcohol and drugs was highest among American Indians/Alaskan Natives and lowest among Asian/Native Hawaiian/Pacific Islander men and Hispanic women. Whites, Blacks, and Hispanics had intermediate levels of co-use.

### Prevalence of AUDs, DUDs, and Co-Morbidity

Approximately 8.5 percent of adults in the United States had an AUD and 2 percent had a DUD during the past year (table 2). Moreover, 1.1 percent of all adults had a co-morbid AUD and DUD, representing approximately 2.5 million adults. As with alcohol and drug use, these data indicate that more than half of people with a DUD also had an AUD, whereas only about one-eighth of people with an AUD had a DUD.

#### Prevalence by Gender

Men, compared with women, had approximately 2.5 times higher rates of any AUD, any DUD, and co-morbid disorders (table 2) as well as AUDs only (10.7 vs. 4.3 percent, respectively^9^). Men also had higher rates of DUDs only (1.1 vs. 0.7 percent, respectively^10^).

#### Prevalence by Age-Group

**Figure 2:**
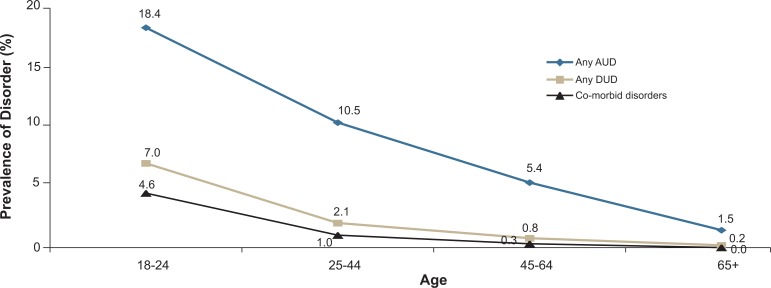
Prevalence (%) of any past-year alcohol use disorder (AUD), drug use disorder (DUD), and co-morbid disorders by age in the United States, 2001–2002 NESARC. NOTE: Data are drawn from Table 2.

The prevalence of AUDs, DUDs, and co-morbid disorders decreased with age (figure 2), with the highest rates (about 18 percent) found among the youngest group and the lowest rates found among the oldest group.^11^ In terms of an absolute percentage decrease, the sharpest drop for all disorder categories occurred between the group of 18- to 24-year-old subjects and the group of 25- to 44-year-old subjects.

#### Prevalence by Race/Ethnicity

American Indians/Alaskan Natives displayed the highest prevalence of AUDs, DUDs, and co-morbid disorders (about 12 percent, 5 percent, and 4 percent, respectively), and Asians/Native Hawaiians/Pacific Islanders had the lowest rates (4.5 percent, 1.4 percent, and 0.7 percent, respectively) (table 2). The rank ordering of DUDs generally followed that of AUDs, with the exception of Blacks, who had the second highest rate of DUDs, yet had almost the lowest rate of AUDs. Co-morbidity rates generally displayed little variability across racial/ethnic groups, except for that of American Indians/ Alaskan Natives, which were significantly higher than those of the other groups.

### Prevalence of Drug Use, Weekly Drug Use, and DUDs by Drinking Characteristics

**Figure 3:**
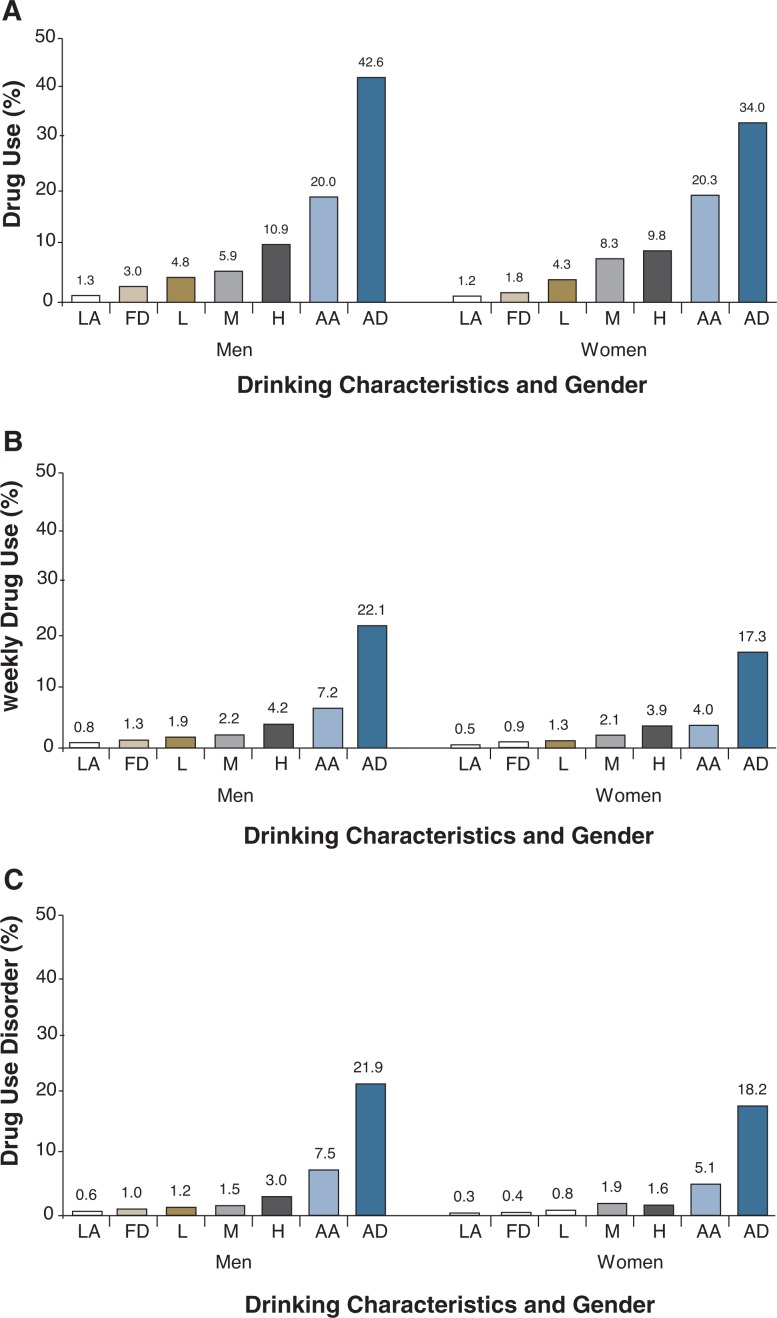
**A)** Prevalence of past-year drug use by past-year drinking characteristics and gender in the United States, 2001–2002 NESARC. **B)** Prevalence of past-year weekly drug use by past-year drinking characteristics and gender in the United States, 2001–2002 NESARC. **C)** Prevalence of past-year drug use disorder by past-year drinking characteristics and gender in the United States, 2001–2002 NESARC. NOTE: Data are drawn from Table 3. AA = Alcohol abuse; AD = Alcohol dependence; FD = Former drinker; H = Heavy drinker; L = Light drinker; LA = Lifetime abstainer; M = Moderate drinker.

NESARC data also demonstrate how drug use is related to the characteristics of alcohol consumption (i.e., lifetime abstainer; former drinker; light, moderate, or heavy drinker; alcohol abuse; and alcohol dependence). For all three drug measures—any drug use, weekly drug use, and any DUD—the rates increased monotonically with increasing levels of alcohol consumption and the presence of alcohol abuse and dependence (table 3, figures 3A–C). For instance, among both men and women, the prevalence of any drug use was lowest (i.e., around 1 percent) among lifetime alcohol abstainers, increased with levels of alcohol consumption, and peaked among alcohol-dependent people (about 43 percent for men and about 34 percent for women, respectively) (figure 3A). Similar positive trends also were observed between drinking characteristics and weekly drug use (figure 3B) or any DUD (figure 3C). The association between alcohol dependence and the three drug use measures was particularly strong compared with the other drinking levels and alcohol abuse. For example, alcohol-dependent men and women had much higher prevalence of drug use than lifetime alcohol abstainers, representing a 33 and 28 times increased risk for drug use, respectively.

### Prevalence of Drug-Specific Drug Use and DUDs among Individuals with AUDs

Approximately 29 percent of people with AUDs also had used other drugs (table 4). However, this estimate varied considerably by drug type. For instance, cannabis use was by far the most commonly associated with AUDs (i.e., about one-quarter of cannabis users also had an AUD). The co-use of other drugs was less common, ranging from about 9.5 percent for opioids to less than 1 percent for inhalant/solvent, heroin, or other drugs. Few gender differences were apparent, except that cannabis use was more prevalent among men than women.

About one-eighth of people with an AUD also had a DUD (table 4). Similar to the pattern seen with drug use, rates of drug-specific DUDs varied considerably by drug type, with the highest for cannabis (about 10 percent) and much lower ones for the rest of drugs. No notable gender differences were evident, except that co-morbid cannabis use disorder was more prevalent among men than women.

## Discussion and Conclusion

This study presents epidemiologic data on alcohol and drug co-use and co-morbidity in the adult population of the United States. With approximately 12.6 million American adults having used both alcohol and other drugs in the past year, the extent of co-use is quite high. The number for co-morbid disorders is considerably lower, yet still sizable, with approximately 2.5 million American adults having both an AUD and a DUD. Consistent with other studies (Burns and Teeson 2002; Grant and Pickering 1996; Regier et al. 1990; Stinson et al. 2005), the prevalence of alcohol use and AUDs are much higher among people with drug use and DUDs, respectively, than vice versa. This reflects the fact that alcohol is much more widely used than other drugs. Also, as suggested by some studies, alcohol use may be a gateway to the use of illegal or more potent drugs (Wagner and Anthony 2002).

Furthermore, higher rates of co-use and co-morbidity were found among certain demographic subgroups. For example, the current analysis revealed that the prevalence of co-use and co-morbidity was higher in men than women. Moreover, the highest rates were seen in the youngest age-groups, with a steady decline observed in older age-groups. Among the racial/ ethnic subpopulations, American Indians/Alaskan Natives had the highest rates of alcohol and drug co-use and co-morbidity among both men and women. This last finding is particularly important but rarely reported in the literature. Overall, the higher rates of co-use and co-morbidity found in these demographic subgroups were not surprising, given reports in the literature of higher rates of alcohol use, drug use, AUDs, and DUDs among these subpopulations (NIAAA 1998; Substance Abuse and Mental Health Services Administration [SAMSHA] 2007). Results from the current study suggest that prevention and treatment strategies for co-morbid alcohol and drug use disorders should place a special emphasis on men and focus efforts on other high-risk groups, such as young adults and American Indians/Alaskan Natives.

In addition to quantifying the current rates of co-use and co-morbidity of alcohol and other drugs in the United States, the present study was unique in its investigation of the association between drinking characteristics and any drug use and DUD. Results showed a dose-response relationship between the two classes of substances, with rates of drug use, weekly drug use, and DUDs increasing monotonically with increasing levels of alcohol consumption and the presence of AUDs. This dose-response relationship is consistent with studies that have examined specific classes of drugs. McCabe and colleagues (2006), using NESARC, found that the prevalence of any past-year prescription drug use (i.e., opioid, sedative, tranquilizer, or stimulant use) was lowest among past-year alcohol abstainers (about 1 percent) and increased to approximately 2 percent among non–binge drinkers (without an AUD), 4 percent among binge drinkers (without an AUD), 8 percent among alcohol abusers, and 22 percent among those with alcohol dependence. Similarly, in a nationally representative Australian study, Degenhardt and Hall (2003) reported that the prevalence of past-year cannabis use increased monotonically by drinking levels, ranging from approximately 2 percent among past-year abstainers to 7 percent among non-AUD drinkers, 27 percent among alcohol abusers, and 32 percent among those with alcohol dependence. In addition, Degenhardt and Hall documented that the rates of past-year cannabis use disorders increased monotonically—ranging from 1 percent to 2, 11, and 15 percent, respectively, for these groups. Furthermore, the findings from the current study are consistent with earlier studies that looked at DUDs in general by AUDs. For example, Kessler and colleagues (1997) and Ross (1995) reported a substantially higher prevalence of DUDs among people with alcohol dependence than people who abuse alcohol.

The present study reproduced findings first presented by Stinson and colleagues (2005) that documented the prevalence of specific DUDs among people with AUDs. These findings indicated that cannabis use disorder was by far the most frequently occurring co-morbid DUD (10 percent), followed by cocaine and opioid use disorders (approximately 2.5 percent and 2.4 percent respectively) and other DUDs (less than 2 percent). Thus, results referring to “any DUD co-morbidity,” as presented in the present study, are disproportionately reflective of cannabis use disorder, as opposed to other co-morbid DUDs. The magnitudes of these specific co-morbidity rates are similar to those found in a recent study that used data from the Australian National Survey of Mental Health and Well Being (Burns and Teeson 2002). In addition to the aforementioned studies, the present study was unique in its examination of how rates of specific co-morbid DUDs vary by gender. These results revealed few gender differences, with the exception of higher rates of co-morbid cannabis use disorder among men than women. The present study also expanded upon the work of Stinson and colleagues (2005) by presenting rates of drug-specific drug use among adults with AUDs. Similar to the pattern seen with drug-specific DUDs, cannabis was the drug most frequently used among people with AUDs (approximately 23 percent).

Thus, the prevalence estimates presented in this study provide health care policymakers and treatment planners with a comprehensive assessment of the state of the use, co-use, and co-morbidity of alcohol and other drugs in the United States. Nonetheless, the current study is descriptive in nature. Given the high rates of co-use and co-morbidity in this country, continued research into the mechanisms underlying co-use and co-morbidity certainly are warranted.

## Figures and Tables

**Table 1:** Prevalence (%) of Past-Year Alcohol and Drug Use and Co-Use in the United States, by Gender, Age, and Race/Ethnicity, 2001–2002 NESARC

	**Alcohol/Drug Use**							
	**No Use**		**Alcohol Use**		**Any Drug Use**		**Co-Use**	
	**%**	95% CI*	**%**	95% CI*	**%**	95% CI*	**%**	95% CI*

**Total**	34.0	33.4–34.5	65.4	64.8–66.0	6.2	5.9–6.5	5.6	5.3–5.9
**Men**	27.5	26.7–28.4	71.8	71.0–72.6	7.8	7.3–8.3	7.1	6.7–7.6
**Age**								
18–24	24.3	22.2–26.5	74.8	72.6–76.9	20.3	18.3–22.3	19.4	17.4–21.4
25–44	21.6	20.4–22.9	77.7	76.4–78.9	8.7	7.9–9.5	8.0	7.2–8.9
45–64	29.0	27.6–30.5	70.4	68.9–71.9	4.2	3.5–4.8	3.6	3.0–4.2
65+	44.2	42.2–46.2	55.4	53.3–57.4	1.2	0.8–1.5	0.7	0.4–1.0
**Race/Ethnicity**								
White	25.2	24.2–26.2	74.3	73.3–75.3	7.9	7.3–8.5	7.4	6.8–8.0
Black	36.6	34.5–38.7	62.6	60.5–64.8	8.7	7.3–10.1	7.9	6.6–9.2
American Indian/Alaskan Native	32.7	25.–939.5	65.5	58.6–72.3	10.8	7.2–14.4	9.0	5.7–12.2
Asian/Native Hawaiian/Pacific Islander	37.7	33.1–42.4	61.5	56.8–66.2	5.9	3.7–8.0	5.1	3.0–7.2
Hispanic	29.1	26.9–31.3	70.0	67.7–72.2	6.4	5.4–7.5	5.5	4.5–6.5
**Women**	39.9	39.1–40.6	59.6	58.8–60.4	4.8	4.4–5.1	4.2	3.9–4.5
**Age**								
18–24	32.7	30.6–34.8	66.8	64.7–69.0	13.0	11.5–14.5	12.5	11.0–14.0
25–44	31.1	29.9–32.3	68.3	67.1–69.5	5.6	5.0–6.1	5.0	4.4–5.5
45–64	40.9	39.4–42.3	58.5	57.1–60.0	2.6	2.1–3.0	2.0	1.6–2.3
65+	61.8	60.3–63.4	37.6	36.0–39.2	1.0	0.7–1.4	0.5	0.2–0.7
**Race/Ethnicity**								
White	34.4	33.5–35.4	65.1	64.1–66.1	4.8	4.4–5.3	4.4	4.0–4.8
Black	53.5	51.8–55.2	45.9	44.2–47.7	4.3	3.6–5.0	3.7	3.1–4.4
American Indian/Alaskan Native	46.8	40.2–53.4	51.7	45.1–58.2	8.5	5.2–11.8	7.0	4.3–9.7
Asian/Native Hawaiian/Pacific Islander	62.7	58.6–66.8	36.1	32.0–40.2	4.7	2.8–6.5	3.5	1.9–5.1
Hispanic	49.6	47.5–51.6	49.5	47.4–51.6	4.0	3.2–4.7	3.0	2.3–3.7

* 95% CI = 95% confidence interval for the estimated percentage.

Note: The prevalence of “alcohol use only” and “drug use only” is derived as follows: % alcohol use only = % any alcohol use – % co-use; % drug use only = % any drug use – % co-use.

**Table 2:** Prevalence (%) of Past-Year DSM–IV Alcohol Use Disorder (AUD), Drug Use Disorder (DUD), and Co-Morbid Disorders in the United States, by Gender, Age, and Race/Ethnicity, 2001–2002 NESARC

	**Alcohol/Drug Use Disorder**							
	**No Disorder**		**Any AUD**		**Any DUD**		**Co-morbid**	
	**%**	95% CI*	**%**	95% CI*	**%**	95% CI*	**%**	95% CI*

**Total**	90.7	90.3–91.0	8.5	8.1–8.8	2.0	1.8–2.2	1.1	1.0–1.2
Gender								
Men	86.5	85.9–87.1	12.4	11.7–13.0	2.8	2.5–3.1	1.7	1.4–2.0
Women	94.4	94.1–94.8	4.9	4.5–5.2	1.2	1.1–1.4	0.6	0.4–0.7
Age								
18–24	79.1	77.7–80.5	18.4	17.0–19.7	7.0	6.2–7.9	4.6	3.8–5.3
25–44	88.5	87.9–89.2	10.5	9.9–11.0	2.1	1.8–2.3	1.0	0.9–1.2
45–64	94.1	93.6–94.6	5.4	5.0–5.9	0.8	0.6–1.0	0.3	0.2–0.4
65+	98.4	98.1–98.7	1.5	1.1–1.8	0.2^†^	0.1–0.3	0.0^†^	0.0–0.0
Race/Ethnicity								
White	90.2	89.8–90.7	8.9	8.5–9.4	1.9	1.7–2.1	1.1	0.9–1.2
Black	91.9	91.1–92.7	6.9	6.1–7.6	2.4	1.9–2.8	1.1	0.8–1.4
American Indian/Alaskan Native	86.5	83.5–89.5	12.1	9.2–15.0	4.9	3.1–6.7	3.5	1.9–5.1
Asian/Native Hawaiian/Pacific Islander	94.7	93.3–96.1	4.5	3.3–5.8	1.4^†^	0.6–2.2	0.7^†^	0.2–1.2
Hispanic	91.4	90.5–92.3	7.9	7.1–8.8	1.7	1.3–2.2	1.1	0.7–1.4

* 95% CI = 95% confidence interval for the estimated percentage.

† Relative standard error >0.30. The relative standard error is a measure of the unreliability of the prevalence estimate, calculated by dividing the standard error by the value of the prevalence estimate. A relative standard error >0.30 is not considered statistically reliable, and the prevalence estimate should be interpreted with caution.

Note: The prevalence of “AUD only” and “DUD only” is derived as follows: % AUD only = % any alcohol use disorder – % co-use; % DUD = % any drug use disorder – % co-morbid.

**Table 3:** Prevalence (%) of Any Past-Year Drug Use, Weekly Drug Use, and Drug Use Disorder (DUD) by Drinking Characteristics and Alcohol Use Disorders (AUDs) in the United States, 2001–2002 NESARC

	**Any Drug Use**		**Weekly Drug Use**		**Any DUD**	
	**%**	95% CI*	**%**	95% CI*	**%**	95% CI*

**Total**	6.2	5.9–6.5	2.5	2.3–2.7	2.0	1.8–2.2
**Men**	7.8	7.3–8.3	3.4	3.0–3.7	2.8	2.5–3.1
Lifetime abstainer	1.3	0.8–1.8	0.8	0.4–1.2	0.6	0.3–0.9
Former drinker	3.0	2.2–3.7	1.3	0.9–1.7	1.0	0.5–1.4
Current drinker	9.9	9.3–10.6	4.2	3.8–4.7	3.6	3.2–4.0
Light	4.8	4.1–5.4	1.9	1.5–2.3	1.2	0.9–1.5
Moderate	5.9	4.9–6.9	2.2	1.6–2.9	1.5	1.0–2.1
Heavy	10.9	8.6–13.2	4.2	3.0–5.5	3.0	1.8–4.2
Any AUD	29.9	27.5–32.3	13.7	11.9–15.6	13.8	11.9–15.6
Alcohol abuse	20.0	17.3–22.7	7.2	5.5–8.8	7.5	5.9–9.0
Alcohol dependence	42.6	38.6–46.6	22.1	18.7–25.5	21.9	18.4–25.3
**Female**	4.8	4.4–5.1	1.7	1.5–1.9	1.2	1.1–1.4
Lifetime abstainer	1.2	0.8–1.5	0.5	0.3–0.7	0.3	0.1–0.5
Former drinker	1.8	1.3–2.3	0.9	0.6–1.3	0.4	0.2–0.6
Current drinker	7.0	6.5–7.5	2.4	2.1–2.6	1.9	1.6–2.1
Light	4.3	3.8–4.7	1.3	1.0–1.5	0.8	0.6–1.0
Moderate	8.3	6.5–10.0	2.1	1.2–3.0	1.9	1.0–2.7
Heavy	9.8	7.7–11.8	3.9	2.6–5.2	1.6	0.6–2.6
Any AUD	26.8	23.7–29.8	10.3	8.1–12.5	11.4	9.2–13.5
Alcohol abuse	20.3	16.4–24.1	4.0	2.3–5.7	5.1	3.2–7.0
Alcohol dependence	34.0	29.1–38.8	17.3	13.3–21.3	18.2	14.3–22.1

* 95% CI = 95% confidence interval for the estimated percentage.

**Table 4:** Prevalence (%) of Past-Year Drug-Specific Drug Use and Drug Use Disorders (DUDs) Among Adults With a Past-Year Alcohol Use Disorder in the United States, 2001–2002 NESARC

	**Drug Use**						**DUDs**					

	**Total**		**Men**		**Women**		**Total**		**Men**		**Women**	
					
	**%**	95% CI*	**%**	95% CI*	**%**	95% CI*	**%**	95% CI*	**%**	95% CI*	**%**	95% CI*
Any	29.0	27.1–30.9	29.9	27.5–32.3	26.8	23.7–29.8	13.1	11.7–14.4	13.8	11.9–15.6	11.4	9.2–13.5
Cannabis	22.5	20.7–24.2	23.9	21.6–26.2	19.1	16.3–21.9	9.9	8.6–11.1	10.9	9.3–12.6	7.5	5.7–9.3
Opioid	9.5	8.2–10.8	9.6	7.9–11.3	9.3	7.3–11.3	2.4	1.7–3.1	2.7	1.8–3.7	1.7	0.9–2.5
Sedative	5.5	4.5–6.5	5.5	4.2–6.7	5.5	3.9–7.0	0.8^†^	0.5–1.1	0.6^†^	0.2–1.0	1.0^†^	0.4–1.6
Cocaine	4.7	3.8–5.6	5.0	3.8–6.2	4.0	2.6–5.4	2.5	1.8–3.2	2.8	1.8–3.7	1.9	1.1–2.8
Hallucinogen	4.7	3.8–5.6	4.8	3.6–5.9	4.4	2.9–5.9	1.3	0.8–1.8	1.2	0.7–1.8	1.5	0.7–2.4
Tranquilizer	4.6	3.7–5.5	4.8	3.6–5.9	4.2	2.8–5.5	0.9	0.5–1.2	0.9	0.4–1.3	0.8^†^	0.2–1.4
Amphetamine	3.3	2.6–4.0	2.7	1.9–3.6	4.6	3.1–6.1	1.2	0.8–1.7	0.8	0.4–1.2	2.2	1.2–3.3
Inhalant/ Solvent	0.9	0.5–1.4	1.1	0.5–1.6	0.6^†^	0.0–1.2	0.2^†^	0.0–0.4	0.2^†^	−0.1–0.5	0.1^†^	0.0–0.2
Heroin	0.4^†^	0.1–0.7	0.3^†^	0.1–0.5	0.5^†^	−0.2–1.2	0.3^†^	0.0–0.6	0.2^†^	0.0–0.4	0.5^†^	−0.2–1.2
Other	0.0^†^	0.0–0.0	0.0^†^	−0.1–0.1	0.0^†^	0.0–0.0	0.0^†^	0.0–0.0	0.0^†^	−0.1–0.1	0.0^†^	0.0–0.0

* 95% CI = 95% confidence interval for the estimated percentage.

† Relative standard error >0.30. The relative standard error is a a measure of the unreliability of the prevalence estimate, calculated by dividing the standard error by the value of the prevalence estimate. A relative standard error >0.30 is not considered statistically reliable, and the prevalence estimate should be interpreted with caution.

## Footnotes

1 Co-morbidity estimates derived from past-year diagnoses also may capture noncontemporaneous AUDs and DUDs; however, this is much less likely to occur with co-morbidity estimates derived from lifetime diagnoses.

2 The definitions of light, moderate, and heavy drinkers are consistent with those put forth in the *Dietary Guidelines for Americans* published by the U.S. Department of Health and Human Services and U.S. Department of Agriculture (2005).

3 Includes the nonprescribed use of analgesics such as codeine and percodan.

4 Although heroin technically is considered a member of the opioid class of drugs, NESARC collects separate data on its use, abuse, and dependence.

5 See Falk et al. (2006) for an in-depth look at the co-occurrence of AUDs and nicotine dependence.

6 The standard error is a measure of the precision of a given estimate and can be used to determine whether two estimates are significantly different from each other. It reflects the variability in the estimate that can be expected if multiple measurements are made on random samples drawn from the same population.

7 Derived from data in Table 1 by subtracting percent co-use from percent any drug use.

8 Derived from data in Table 1 by subtracting percent co-use from percent any alcohol use.

9 Derived from data in Table 2 by subtracting percent co-use from percent any alcohol use.

10 Derived from data in Table 2 by subtracting percent co-use from percent any AUD.

11 The prevalence estimates for this age-group are not statistically reliable, however, because of the small cell sizes and should thus be interpreted with caution.

## References

[b1-arh-31-2-100] American Psychiatric Association (APA) (1994). Diagnostic and Statistical Manual of Mental Disorders, Fourth Edition (DSM–IV).

[b2-arh-31-2-100] Angold A, Costello EJ, Erkaneli A (1999). Co-morbidity. Journal of Child Psychology and Psychiatry.

[b3-arh-31-2-100] Burns L, Teesson M (2002). Alcohol use disorders co-morbid with anxiety, depression and drug use disorders: Findings from the Australian National Survey of Mental Health and Well Being. Drug and Alcohol Dependence.

[b4-arh-31-2-100] Degenhardt L, Hall W (2003). Patterns of co-morbidity between alcohol use and other substance use in the Australian population. Drug and Alcohol Review.

[b5-arh-31-2-100] Falk DE, Yi H, Hiller-Sturmhöfel S (2006). An epidemiologic analysis of co-occurring alcohol and tobacco use and disorders: Findings from the National Epidemiologic Survey on Alcohol and Related Conditions. Alcohol Research & Health.

[b6-arh-31-2-100] Grant BF, Pickering RP (1996). Co-morbidity between DSM-IV alcohol and drug use disorders: Results from the National Longitudinal Alcohol Epidemiologic Survey. Alcohol Health & Research World.

[b7-arh-31-2-100] Grant BF, Dawson DA, Hasin DS (2001). The Alcohol Use Disorder and Associated Disabilities Interview Schedule–DSM-IV Version.

[b8-arh-31-2-100] Grant BF, Kaplan K, Shepard J, Moore T (2003). Source and Accuracy Statement for Wave 1 of the 2001-2002 National Epidemiologic Survey on Alcohol and Related Conditions.

[b9-arh-31-2-100] Grant BF, Dawson DA, Stinson FS (2004). The 12-month prevalence and trends in DSM-IV alcohol abuse and dependence: United States, 1991–1992 and 2001–2002. Drug and Alcohol Dependence.

[b10-arh-31-2-100] Helzer JE, Pryzbeck TR (1988). The co-occurrence of alcoholism with other psychiatric disorders in the general population and its impact on treatment. Journal of Studies on Alcohol.

[b11-arh-31-2-100] Kessler RC, Crum RM, Warner LA (1997). Lifetime co-occurrence of DSM-III-R alcohol abuse and dependence with other psychiatric disorders in the National Co-morbidity Survey. Archives of General Psychiatry.

[b12-arh-31-2-100] Klein RJ, Proctor SE, Boudreault MA, Turczyn KM (2002). Healthy People 2010 criteria for data suppression. Healthy People 2010 Statistical Notes.

[b13-arh-31-2-100] McCabe SE, Cranford JA, Boyd CJ (2006). The relationship between past-year drinking behaviors and nonmedical use of prescription drugs: Prevalence of co-occurrence in a national sample. Drug and Alcohol Dependence.

[b14-arh-31-2-100] National Institute on Alcohol Abuse and Alcoholism (NIAAA) (1998). Drinking in the United States: Main Findings from the 1992 National Longitudinal Alcohol Epidemiologic Survey (NLAES). U.S. Alcohol Epidemiologic Data Reference Manual, Volume 6, First Edition.

[b15-arh-31-2-100] Petrakis IL, Gonzalez G, Rosenheck R (2002). Comorbidity of alcoholism and psychiatric disorders: An overview. Alcohol Research & Health.

[b16-arh-31-2-100] Regier DA, Farmer ME, Rae DS (1990). Co-morbidity of mental disorders with alcohol and other drug abuse. Results from the Epidemiologic Catchment Area (ECA) Study. JAMA: Journal of the American Medical Association.

[b17-arh-31-2-100] Research Triangle Institute (2004). SUDAAN Language Manual, Release 9.0.

[b18-arh-31-2-100] Ross HE (1995). DSM-III-R alcohol abuse and dependence and psychiatric co-morbidity in Ontario: Results from the Mental Health Supplement to the Ontario Health Survey. Drug and Alcohol Dependence.

[b19-arh-31-2-100] Stinson FS, Grant BF, Dawson DA (2005). Co-morbidity between DSM-IV alcohol and specific drug use disorders in the United States: Results from the National Epidemiologic Survey on Alcohol and Related Conditions. Drug and Alcohol Dependence.

[b20-arh-31-2-100] Substance Abuse and Mental Health Services Administration (SAMHSA) (2007). Substance use and substance use disorders among American Indians and Alaska Natives. http://www.oas.samhsa.gov/2k7/AmIndians/AmIndians.pdf.

[b21-arh-31-2-100] U.S. Department of Health and Human Services and U.S. Department of Agriculture (2005). Dietary Guidelines for Americans, 2005.

[b22-arh-31-2-100] Wagner FA, Anthony JC (2002). Into the world of illegal drug use: Exposure opportunity and other mechanisms linking the use of alcohol, tobacco, marijuana, and cocaine. American Journal of Epidemiology.

